# Impact of exercise changes on body composition during the college years - a five year randomized controlled study

**DOI:** 10.1186/s12889-016-2692-y

**Published:** 2016-01-19

**Authors:** Wolfgang Kemmler, Simon von Stengel, Matthias Kohl, Julia Bauer

**Affiliations:** 1 Institute of Medical Physics, University of Erlangen-Nürnberg, Henkestrasse 91, 91052 Erlangen, Germany; 2 Department of Medical and Life Sciences, University of Furtwangen, Jakob-Kienzle-Str. 17, 78054 Schwenningen, Germany; 3 Institute of Dentistry, University-Hospital, Erlangen-Nürnberg, Glückstrasse 11, 91054 Erlangen, Germany

**Keywords:** Fat mass, Physical fitness, Lean body mass, Physical activity, Young adults, Students

## Abstract

**Background:**

Observational studies have consistently reported severe weight gains during the college years; information about the effect on body composition is scarce, however. Thus, the aim of the study was to determine the effect of exercise changes on body composition during 5 years at university.

**Methods:**

Sixty-one randomly selected male and female dental (DES; 21 ± 3 years., 22 ± 2 kg/m^2^) and 53 sport (physical education) students (SPS; 20 ± 2 years., 22 ± 3 kg/m^2^) were accompanied over their 5-year study program. Body mass and body composition as determined via Dual-Energy x-ray-absorptiometry (DXA) at baseline and follow-up were selected as primary study endpoints. Confounding parameters (i.e., nutritional intake, diseases, medication) that may affect study endpoints were determined every two years. Endpoints were log-transformed to stabilize variance and achieve normal distributed values. Paired t-tests and unpaired Welch-t-tests were used to check intra and inter-group differences.

**Results:**

Exercise volume decreased significantly by 33 % (*p* < .001) in the DES and increased significantly (*p* < .001) in the SPS group. Both cohorts comparably (*p* = .214) gained body mass (SPS: 1.9 %, 95 %-CI: 0.3−3.5 %, *p* = .019 vs. DES: 3.4 %, 1.4−5.5 %, *p* = .001). However, the increase in the SPS group can be completely attributed to changes in LBM (2.3 %, 1.1−3.5 %, *p* < 0.001) with no changes of total fat mass (0.6 %, −5.0−6.5 %, *p* = 0.823), while DES gained total FM and LBM in a proportion of 2:1. Corresponding changes were determined for appendicular skeletal muscle mass and abdominal body-fat. Maximum aerobic capacity increased (*p* = .076) in the SPS (1.6 %, −0.2−3.3 %) and significantly decreased (*p* = .004) in the DES (−3.3 %, −5.4 to −1.2 %). Group differences were significant (*p* < .001). With respect to nutritional intake or physical activity, no relevant changes or group differences were observed.

**Conclusion:**

We conclude that the most deleterious effect on fatness and fitness in young college students was the pronounced decreases in exercise volume and particularly exercise intensity.

**Trial registration:**

NCT00521235; “Effect of Different Working Conditions on Risk Factors in Dentists Versus Trainers. A Combined Cross sectional and Longitudinal Trial with Student and Senior Employees.”; August 24, 2007.

## Background

Transition from (high) school and home to apprenticeship, university, civil or military service along with moving to a new unfamiliar setting [1–3] induce pronounced changes of lifestyle [4–6] that can severely challenge a subject’s health status [5, 6]. With respect to body composition, the 5.5-times higher weight gain of college students compared with the general population [7], which can be largely attributed to increases of fat mass [8], may be the most prominent negative consequence of this new situation. The main reason for this development may be the severe decline of physical activity as a protective factor that was reported to be far above average during this period of life [3, 9]. Although this problem was primarily reported for US student cohorts, the general problem of drastically reduced physical activity combined with unhealthy life style changes may concern many young adults. Maintaining or increasing the amount of sport and/or physical exercise may be the most effective tool in fighting overweight and obesity in this period of life. Thus, the aim of the study was to determine the − preferably − isolated effect of physical activity, or, more specifically, exercise on the development of body composition during young adulthood. In order to achieve this goal we accompanied two cohorts of students with fundamentally different exercise patterns (sports vs. dentistry students), but comparable basic condition, setting and situation before and during their study course of ≈ 5 years. The rational behind selecting these cohorts was based on the assumption that the new unfamiliar setting and the high demands related to the dentistry college course will significantly decrease exercise levels and thus dentistry students may be ideal representatives for corresponding life style changes among young adults. On the other hand, sports students are one of the few cohorts to increase or at least maintaine their former exercise levels. Thus, we expect that dentistry and sports student may be most suitable to determine the effect of occupational related sports and exercise increases or reductions on body composition during young adulthood.

Our primary hypothesis was that 5-year changes in (a) total body fat mass and (b) lean body mass would differ significantly between sports/physical education) students (SPS) and dentistry students (DES) with no significant differences with respect to body mass gain.

Our secondary hypothesis was that 5-year changes in (a) appendicular skeletal muscle mass, (b) abdominal fat mass and (c) aerobic capacity would differ significantly between SPS and DES.

## Methods

### Study design

The present study was a randomized, semi-blinded (i.e., researchers and assessors were blinded) 5-year study that determined the effect of exercise (reduction) on health risk factors with particular consideration of body composition during the college years. The study was part of the project “Effect of Different Working Conditions on Risk Factors in Dentists Versus Trainers. A Combined Cross sectional and Longitudinal Trial With Student and Senior Employees”, that was conducted from May 2007 through December 2013 by the Institute of Medical Physics, Friedrich Alexander-University Erlangen-Nuremberg (FAU), Germany. The study was registered under www.clinicaltrials.gov (NCT00521235).

### Study population, setting

**Fig. 1:**
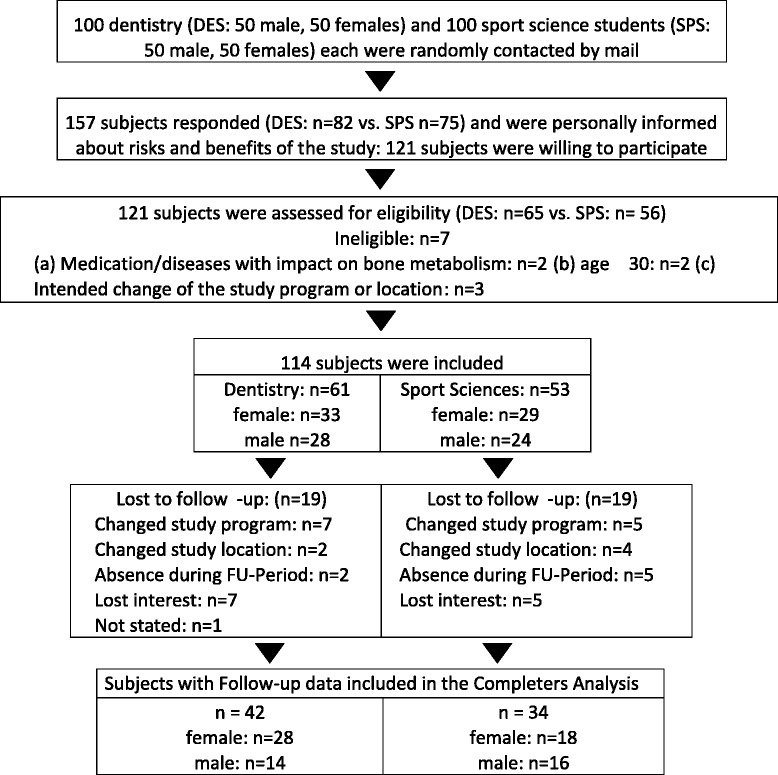
“Flow-Chart” of the study

The study took place at the Friedrich Alexander-University Erlangen-Nuremberg (FAU), Bavaria, Germany between November 2007 and July 2013. Study participants have been extensively described in a recent article on changes of Peak Bone Mass in this cohort [10], thus only a brief description along with a recapitulated flow chart (Fig. 1) will be presented here.

#### Inclusion/exclusion criteria

Based on our exclusion criteria of (a) intended change of study program or study location, (b) age ≥30 years, (c) diseases/medication affecting body composition, (d) pregnancy, 114 out of 157 subjects who responded proved to be eligible and were willing to participate (DES: *n* = 61 vs. SPS: *n* = 53). Figure 1 shows the participant flow through the study course.

#### Randomization

Our randomization strategy was based on lists of young adults embarking on dentistry or sport sciences degrees in 2007 and 2008 provided by the university administration. One hundred starters in each in the disciplines “dentistry” (50 male, 50 female) and “sport sciences” (50 male, 50 female) were selected by a computer-generated random list and contacted by post.

### Outcome

#### Primary study outcome

- Total Body Fat Mass (tFM)
- Total Body Lean Mass (LBM)

#### Secondary/experimental endpoints

- Appendicular Skeletal Muscle Mass (ASMM)
- Abdominal Fat Mass (aFM)
- Aerobic capacity (VO_2_peak)
- Body mass

### Testing procedures

Baseline tests were performed ≤ 6 weeks after the start of the study program and during the final semester/first months of the school internship (SPS), or during the semester break between the 9^th^ and 10^th^ semesters (DES) in order to adjust for the longer study period of the DES. Thus, the observation period averaged 4.8 ± 0.5 years for both groups.

Subjects were consistently tested at the same time of the day (±2 h) and by the same researcher. Assessments were carried out in a fixed order and in a blinded fashion, which means that researchers and assessors were not informed about the status of the subjects (DES or SPS) and were not allowed to ask questions of that nature.

### Operational definitions, assessment tools

#### Anthropometry

Height was determined with a stadiometer (Holtain Ltd, Crymych, UK). Additionally, body composition was measured with minimal clothing using Dual-Energy X-Ray Absorptiometry (DXA) (QDR 4500, discovery upgrade, Hologic Inc., Bedford, USA) at study start and at the end of the study period, as per the whole body standard protocol specified by the manufacturer. Region of interest (ROI) for abdominal body fat was determined between the lower edge of the 12^th^ rib and the upper edge of the iliac crest. Appendicular skeletal muscle mass index (fat- and bone-free proportion of the legs and arms [kg] as assessed by DXA / height [m^2^]: ASSM) was segmented and calculated according to the method described by Baumgartner et al. [11].

#### Aerobic capacity

VO_2_, CO_2_ and VE were continuously determined breath by breath using an Oxycon mobile open spirometric system (Viasys, Conshohocken, PA, USA) during a stepwise bicycle ergometer test (3 min, 30 Watt steps; start at 100 Watt) up to a voluntary maximum.

#### Questionnaires

The questionnaires covered several topics: (a) living conditions and social status of the participants and their parents, (b) medical conditions, health status, pain frequency and intensity at different skeletal sites, (c) specific osteoporotic and coronary heart disease risk factors, (d) lifestyle, nutrition, and stimulants. Follow-up questionnaires and structured interviews included several sections of the baseline questionnaire; but the main aim of the FU questionnaire and interview was to control for changes of parameters that may confound our results (e.g., lifestyle, physical activities, medication, diseases).

Baseline physical activity, sports- and exercise levels and their changes during the study course were specifically addressed. History, type, volume and self-rated intensity of physical activity, sports and exercise were determined with the specific questionnaires and personal interviews described and validated in recent publications [12, 13]. Based on this questionnaire, several indices were calculated by three sports scientists (WK, MB, SvS) using the Delphi technique [14]. For this contribution, four indices were applied: (1) A summary of total physical activity (h/week) under consideration of the type and intensity of this activity, rated on a 7-item scale (activity intensity index: AI) (2) The total exercise index (EI, in min/week) defined by weekly frequency x exercise duration per session. (3) In addition, the latter index was structured according to the type of sports and exercise (either aerobic (EI_aer_) or resistance type (EI_res_) exercise (or neither) in min/week). Finally, (4) Both indices (IEI_aer_; IEI_res_) were further rated for their inherent general intensity of the corresponding exercises by multiplying EI_aer/res_ (min/week) × 1 (low), × 2 (moderate), or × 3 (high intensity).

Finally, 4-day dietary protocols were completed in parallel to the questionnaires at baseline, after the 5th semester and at study end. Food consumption was analyzed using the “Freiburger Nutrition Protocol” (nutri-science, Hausach, Germany).

### Intervention

Although we are unable to prescribe or change the corresponding study curriculum, we consider the study program with its obligatory curricula as a profound overall “intervention” which without doubt far exceeds the impact of conventional exercise trials with their limited exercise protocols and participant compliance. Completely unlike conventional trials involving an isolated intervention, starting university affects most aspects of students’ lives.

In order to assess the complete effect of this profound intervention, we tested subjects immediately after the start of their study program and during the last (or penultimate: DES) semester. Since both study protocols have already been described in detail, only a brief characterization of these study programs will be given here.

The regular study period for dentistry is 11 semesters with an average volume of obligatory and facultative lectures, tutorials and required practicals of 32–36 h/week during the semester. Due to obligatory dentistry internships, practical work and key examinations, the occupational workload and stress remained at high levels during the 2-month “semester breaks”. Questionnaires provided after the 5^th^ and 9^th^ semesters to determine the weekly workload directly or indirectly related to the dentistry course revealed an average of 32 ± 9 and 37 ± 12 h/week respectively. However, physical activity during this occupation is relatively low due to the rather immobile sitting and standing positions [15]. In addition to the dentistry-related workload, subjects reported an average 8.8 ± 7.1 h/week of physical activity related to earning a living (paid work), predominately during the semester break.

Sports students (i.e., physical education students) aiming to qualify as secondary-school teachers in Bavaria have to be extremely physically active. Besides the theory component, 1,050 obligatory hours of practical sport are required during the 9-semester study curriculum. Including preparation for practical sport tests and leisure time sports activity or exercise increase this amounts up to 11.9 ± 2.4 h/w. (range: 8–17 h/w.). Obligatory core and facultative disciplines (altogether12 disciplines) included all types of exercises (track and field athletics, swimming, gymnastics, dancing, team and individual ball games, skiing, water sports and martial arts), while core disciplines did not differ for males and females. Due to the preparation for tests and increased leisure time sports activity, the total amount of exercise remained at high levels (i.e., 6 ± 3 h/w.) during the semester breaks. Just like dentistry students, sport students reported additional physical activity due to paid work outside the study program that averaged 9.4 ± 6.2 h/week.

### Statistics

The sample size calculation has been described at length elsewhere [16]. Briefly, we concentrate on the Metabolic Syndrome as a possible consequence of unfavorable changes of body composition the sample size of the study. Our decision to focus on this parameter is based on our estimate that groups differences (pre vs. post per group) for the Metabolic Syndrome Z-Score were less pronounced compared with body fat or lean body mass.

Baseline values are given as means with standard deviations (MV ± SD, Tables 1 and 2). Differences between baseline and follow-up per group were reported as percentage changes (text). The primary and secondary endpoints were log-transformed to stabilize variance and achieve normal distributed values. We used paired t-tests and unpaired Welch-t-tests as appropriate, where all the tests were 2-sided using a significance level of 0.05. SPSS 21.0 (SPSS Inc, Chicago, IL) was used except for the ITT analysis. The procedure of the ITT analysis was described in detail elsewhere [16], thus only a brief description is given. The ITT analysis was performed using the statistics software R (R Development Core Team Vienna, Austria) in combination with multiple imputation by Amelia II [17]. The imputation was repeated 50 times. In addition, we used the approach of Barnard et al. [18] to compute mean, SD (combination of within- and between-imputation variance) and p values (t-distribution with adjusted degrees of freedom).

**Table 1:** Baseline characteristics of male and female sport (SPS) and dentistry students (DES)

Study degree	Sport-Science (SPS)		Dentistry (DES)	
Gender	Female	Male	Female	Male
	*n* = 29	*n* = 24	*n* = 33	*n* = 28
Age [years]	20.1 ± 2.1	20.6 ± 1.9	20.5 ± 2.5	21.1 ± 3.1
Body Mass Index [kg/m^2^]	22.3 ± 3.0^f^	22.2 ± 1.3	20.8 ± 1.6	22.9 ± 2.0
White, caucasian race [%]	97	92	97	89
Direct transfer from home [%]	56	20	52	22
AI[hours/week]^a^	30 ± 10	28 ± 9	32 ± 9	27 ± 11
EI [min/week] ^b^	221 ± 115	270 ± 126	109 ± 65	123 ± 71
IEI [min/week/intensity] ^c^	326 ± 121	437 ± 139	149 ± 76	154 ± 61
VO_2_peak [ml/min/kg] ^d^	46.1 ± 4.2	57.4 ± 5.5	42.2 ± 5.8	52.0 ± 8.8
Energy uptake [MJ/d]	9.43 ± 2.42	10.90 ± 2.51	8.69 ± 1.67	11.12 ± 2.64
Carb./Prot./Fat/Alcohol [%]^e^	59/18/22/1	58/20/18/4	65/14/20/1	57/18/21/4

^a^ Activity Intensity Index; ^b^ Exercise Index (overall exercise volume); ^c^ Intensity Exercise Index for resistance and aerobic type of exercise; calculation of a-c: see methodology section ^d^ as assessed by bicycle ergometry to a voluntary maximum; ^e^ percent of energy intake. ^f^ significantly different from female DES

**Table 2:** Baseline data and overall changes (mean values ± standard deviations) with differences between the groups for Total Body Mass and the primary and secondary study endpoints; sports students (SPS: *n* = 53) vs. dentistry students (DES: *n* = 61)

Study degree	SPS	DES	*p*
Parameters	MV ± SD	MV ± SD	
Total Body Mass [kg] baseline	67.99 ± 8.59	67.90 ± 11.82	.97
Difference after 4.5 years [kg]	69.31 ± 9.03	70.20 ± 9.77	.86
Total Body Fat [kg] baseline	12.58 ± 4.28	14.49 ± 3.73	.01
Difference after 4.5 years [kg]	−0.284 ± 2.245	1.612 ± 3.836	.03
Total Lean Body Mass [kg] baseline	55.37 ± 9.44	53.42 ± 11.73	.33
Difference after 4.5 years [kg]	1.300 ± 2.287	0.863 ± 2.562	.36
Abdominal Body Fat [g] baseline	975 ± 377	1184 ± 449	.01
Difference after 4.5 years [kg]	−14 ± 270	240 ± 498	.02
ASMM [kg]^a^ baseline	25.44 ± 4.97	24.30 ± 6.36	.29
Difference after 4.5 years [kg]	0.589 ± 1.389	0.171 ± 1.859	.23

^a^ Appendicular Skeletal Muscle Mass

### Ethical considerations

The study strictly complies with the WMA Declaration of Helsinki - Ethical Principles for Medical Research Involving Human The ethics committee of the University of Erlangen (Ethik Antrag 3674) and the Bundesamt für Strahlenschutz (Z5-22462/2-2007-041) approved the study protocol. After detailed information, all the study participants signed a written informed consent.

## Results

Table 1 gives the characteristics of both groups for baseline. With the exception of the BMI, baseline values for anthropometric, dietary intake parameters and time living independently/direct move from home did not vary between male SPS vs. DES and female SPS vs. DES. Baseline exercise indices and VO_2_peak, but not (general) physical activity, were significantly higher in SPS males and females compared with DES (Table 1).

Nineteen subjects each per group were lost to follow-up. Reasons for withdrawal were (a) changes of study program or study location outside Bavaria (*n* = 18)); (b) absence during the final FU assessment period (*n* = 7); (c) loss of interest and/or unwilling to accomplish the final FU tests (*n* = 13) (Fig. 1).

### Confounding factors: changes of dietary intake, alcohol consumption and smoking

With one exception, no significant differences were observed after the 5^th^ semester or at study end. Energy uptake increased non-significantly in both groups (SPS: 66 ± 533 kcal vs. DES 41 ± 469 kcal, *p* = .72) with no significant changes of the proportion of macronutrients (*p* ≥ .32). Alcohol consumption doubled in the female cohorts, albeit from a low base, (SPS: 3.3 ± 2.4 to 7.6 ± 6.1 g/d DES: 4.1 ± 3.6 to 7.1 ± 6.1 g/d) and was maintained in the male cohorts. The proportion of smokers among the DES (18 %) and SPS (6 %) group did not significantly change.

### Changes of physical activity, sports and exercise

As given in Table 1, military/civil service, preceding internship or employment led to only 36 % of the SPS and 38 % of the DES immediately moving from home/school to university (females vs. males *p* < .001; Table 1).

With respect to general physical activity, the activity intensity index (AI) increased non-significantly (*p* > .15) by 5-10 % in both groups with no significant group differences at baseline, at study end and with no differences between genders. Changes of AI were related to earning a living.

Baseline values for all exercise indices were significantly higher (*p* ≤ .001) in the SPS compared with the DES, with the most pronounced differences for the specific intensity exercise indices (IEI_res_ and IEI_aer_).

In summary, changes in all the exercise indices differed significantly between groups with significant reductions in the DES versus maintained or increased indices in the SPS (*p* < .001). In detail, leisure time-sports activities and exercise (EI) decreased significantly in the DES group (−34 ± 22 %, *p* = .001) and did not change relevantly in the SPS (−2 ± 13 %, *p* = .36). In this context, the number of subjects who reported exercising ≥2 sessions/week decreased from 38 to 26 subjects in the DES group. However, the most marked reductions (DES) and intergroup difference at follow-up were determined for the intensity exercise indices (IEI). Resistance type IEI decreased by −38 ± 22 % and aerobic IEI decreased by −41 ± 32 % (both *p* < .001), whereas these parameters did not change in the SPS group. Taking into account the fact that these values refer to leisure time exercise only, additional “occupational exercise” arising from the study program described above nearly double the total exercise volume, and further increase exercise complexity and intensity. Thus, exercise was not only maintained in the SPS, but actually increased significantly.

### Primary and secondary endpoints

Since we did not determine significantly different trends for changes of body composition in female versus male DES, or female versus male SPS, we decided to conduct a combined analysis.

Based on comparable baseline values for Body Mass (*p* = .97, Table 2) and Body Mass Index (*p* = .22, Table 1), baseline total and abdominal Fat Mass were significantly higher in the DES compared with the SPS group (tFM: *p* = .013, aFM: *p* < .01), while no significant differences were assessed for baseline Lean Body Mass (*p* = .33) or ASMM (*p* = .29) (Table 2).

Body mass significantly increased in both groups (SPS: 1.9 %, 95 %-CI: 0.3 to 3.5 %, *p* = .02 vs. DES: 3.4 %, 95 %-CI: 1.4 % to 5.5 %, *p* < .001), with no significant difference between groups (*p* = .214); body composition changes did differ widely between groups, however. With respect to body fat the DES-group significantly gained total (10.4 %, 3.3 to 18.1 %, *p* < .01) and abdominal body fat (16.6 %, 5.8 to 28.5 %, *p* < .01) while SPS maintained their total (0.6 %, −5.2 to 6.5 %, *p* = .82) and abdominal body fat −2.6 % (−14.2 to 8.6 %, *p* = 0.63) mass. Corresponding changes for abdominal (*p* = .02) and total body fat mass (*p* = .03) differed significantly between groups.

LBM (SPS: 2.3 %, 1.1 to 3.5 %, *p* < .001 vs. DES: 1.6 %; 0.3 to 2.7 %, *p* = .02) and ASMM (SPS: 2.3 %, 0.7 to 3.9 %, *p* < .01 vs. DES: 0.8 %, −1.0 to 2.8 %, *p* = .37), increased in both groups however, with no significant difference SPS and DES (LBM: *p* = .36; ASMM: *p* = .26). Thus, body mass gain in the SPS group can be completely attributed to changes in LBM with no relevant changes of fat mass, while among the DES group the proportion of fat gain to LBM gain was 2:1.

Based on significant baseline VO_2_peak differences (Table 1), aerobic capacity increased non-significantly (*p* = .08) in the SPS (0.81, 95 %-CI −0.09 to 1.70 ml/min/kg resp. 1.6 %, −0.2 to 3.3 %) and decreased significantly (*p* = .004) in the DES (−1.52, −2.51 to −0.54 ml/min/kg resp. -3.3 %, −5.4 to −1.2 %). Group differences were significant (*p* < .001).

In summary, our hypotheses that refer to Fatness and Fitness can be fully confirmed while the hypotheses that refer to lean body mass (total LBM, ASMM) must be rejected. However, as expected, body mass significantly increased in both groups with no significant differences.

## Discussion

The primary aim of this contribution was to determine the effect of occupational related sports and exercise reductions on body composition. In order to evaluate this issue we looked at a study course with high occupational but low physical activity demands, which makes it very likely that exercise will fall significantly during the study period. We compared this group, which may be a typical representative for the situation facing young adults, with sports students, a cohort with increased or at least maintained exercise levels which were already high at the start.

The first, but not unexpected finding [19] was that both cohorts generated a comparable general physical activity (but not exercise) that increased slightly during the college years. This is in line with (German) data that reported a reduction in sport and exercise participation but not in regular physical activity during young adulthood [19].

Based on high sports ad exercise participation at baseline, DES students reduced their exercise volume by one third during the study period. Furthermore, and potentially even more importantly, exercise intensity for resistance and aerobic type exercise decreased by 40 %. This finding was supported by the observation that the number of DES taking part in competitions decreased by 57 %; with 63 % stating that they competed at a lower level. Due to a lack of data for detailed changes of exercise pattern during young adulthood in Germany (and the US), we are unable to present a full discussion; on the other hand, representative national reports consistently stated that the erosion of sports participation was greatest during this period of life [19, 20].

No relevant changes for dietary intake parameters as determined by dietary records were observed in the present study. Studies that accompany US students during the “freshmen / sophomore period” (review in [4]) or up to senior year [1, 6] predominately reported considerable changes of dietary behavior and pattern, although this did not necessarily imply changes in energy consumption [21, 22].

Considering that other possible covariates (review in [4]) than dietary intake (i.e., ethnicity, baseline BMI, residency, medication, diseases, smoking, alcohol consumption, and general physical activity) did not change either–or at least did not differ between DES and SPS, we largely contribute changes in fitness and fatness to changes in sports and exercise. Some studies which addressed long-term changes of sports and exercise in adolescents and/or young adults support this implication [23–25]. Data of the CARDIA- [25] and HUNT-study [24] which accompanied adolescents (13–19 years) or young adults (18–24 years) over a ≈ 10-year period also confirmed the favorable effect of maintaining/ increasing compared with reductions of exercise on weight or abdominal body fat during young adulthood. However, in line with our finding, maintaining or even increasing “exercise units” did not prevent weight gain (4.3 % and 3.4 % in 5 years) in the CARDIA-cohort [25].

In this context, most studies demonstrated that weight gain during the freshman/sophomore year [21, 26, 27] or the complete college years [8] can be largely attributed to increases of fat mass; however, some studies reported weight gain without changes of fat mass [22, 28]. In the present study both cohorts showed significant weight (i.e., body mass) gain (SPS: 1.9 % vs. DES: 3.4 %), the rate of fat and LBM gains differed significantly between groups, however. While DES gained fat and LBM at a 2:1 rate, body mass changes in the SPS group were completely reflected by LBM changes (Table 2). This finding was supported by data of Crombie et al. [23] which showed significantly more favorable changes of lean (3.2 % vs. -0.2 % vs. 0.2 %) and fat tissue ((−5.2 % vs. 15.4 % vs. 5.8 %) in highly sportive students compared with a less active group of students or army reserve officers during the critical first college semester. Much like the present study, no significant changes among or between groups were observed for energy or macronutrient intake [23]. The timeframe of this study [23] is the freshman period only however, which limits comparability with the present study.

Although non-significant, there are some gender differences with respect to changes of body mass or body composition. Females of both cohorts consistently gained less body mass. Further, female SPS lost more total or abdominal body fat and gained less LBM compared with their male peers. Similarly, female DES gained less body mass and LBM compared with their male counterparts. With respect to other predictors of body mass gain [4] during the early college period, we are unable to confirm the effect of initial BMI, ethnicity or residency.

In line with present data for young adults [29], physical fitness as determined by cardiorespiratory fitness (i.e., VO_2_max, time under load) significantly decreased in male and female DES, while SPS increased their (even high) cardiorespiratory fitness (*p* = .08). Two comparable studies that determined fitness changes in US-military medical students over 2 - [30] or 4 years [31] reported significant decreases of aerobic capacity / cardiorespiratory endurance in their cohorts. However, in contrast to our data (maximum isometric leg extensor strength: −3.5 %, 1.8 to 7.3, *p* < .01) for the DES, body composition and muscular strength were maintained or even increased.

However, general differences with respect to college study in Germany vs. the US as well as some specific features and limitations of the present study may complicate a proper comparison of the data and may explain some of the results of the present study. (a) German students were older and a higher rate of subjects are experienced in independent living thanks to preceding social/military service or education courses. This factor may be important, because potentially lifestyle changes with impact on obesity may happen before the study period. We are unable to address this issue, however most subjects reported no relevant weight changes during this period (b) The DES-group mainly represented a cohort of upperclass children with the excellent grades required to qualify for a demanding university dentistry degree course. Thus, DES students were rather more focused and perhaps less susceptible to lifestyle changes [32]. (c) Although exercise was significantly reduced in the DES group, participation in sports and exercise at study end still exceed the data given for this age group as a whole by far [19, 33]. (d) The SPS group by far exceeded the exercise levels of all active or highly active study groups of corresponding degree courses. However, we opted to compare DES versus SPS due to the predictable changes of sports and exercise parameters generated by the corresponding study curriculum. Due to the “occupational exercise” changes in the SPS, our aim of including a group that preferentially increased or at least maintained its high level of exercise was assured. The demanding study curriculum of the DES, on the other hand, strongly suggested there would be a significant decrease in exercise and sport participation in this cohort. Thus, to a certain degree we hoped to avoid subjective errors or cheating through self-reporting which might otherwise confound our results for nutritional intake, general physical activity and leisure time sports and exercise. (e) Our sample size calculation is based on the Metabolic Syndrome Z-Score as a clinical consequence of unfavorable changes of body composition, which was addressed in another publication [16]. We opted for this parameter on the assumption that differences between the groups were lower compared with lean or fat mass. Thus in summary, the power to address the present issue was not negatively affected by our approach. (f) We are unable to randomly allocate study starters to the different degrees (i.e., SPS vs. DES), thus our strategy scheduled a randomized selection of study starters within the degree courses themselves. (g) We placed a strong emphasis on detecting possible confounders, but some relevant changes may have escaped our attention. (h) Due to the application of DXA-technique with its (albeit rather low) radiation dosage, we decided to abstain from more frequent assessments of body composition parameters in this young cohort.

## Conclusion

In summary, we conclude that the most deleterious effect on fitness and fatness in young college students was closely related to the sharp decreases in exercise volume and—probably to an even higher degree—intensity. The finding that higher general physical activity is obviously unable to compensate for this reduction in exercise, although energy expenditure may be at least the same, supported this conclusion. Since occupation-related reductions of exercise are not restricted to college students but affect most other young adults who move to take up an apprenticeship or a job, a more favorable environment for sport and exercise has to be created by universities or companies. Most effective would be for public health policy to focus on introducing mandatory exercise programs during working hours. Time-effective aerobic and resistance HIT-exercise protocols [34, 35] may be the most feasible and efficient exercise methods for achieving this aim.
